# Fantastic [FeFe]-Hydrogenases and Where to Find Them

**DOI:** 10.3389/fmicb.2022.853626

**Published:** 2022-03-02

**Authors:** Simone Morra

**Affiliations:** Faculty of Engineering, University of Nottingham, Nottingham, United Kingdom

**Keywords:** hydrogenase, energy metabolism, oxygen sensitivity, H-cluster, metalloenzymes

## Abstract

[FeFe]-hydrogenases are complex metalloenzymes, key to microbial energy metabolism in numerous organisms. During anaerobic metabolism, they dissipate excess reducing equivalents by using protons from water as terminal electron acceptors, leading to hydrogen production. This reaction is coupled to reoxidation of specific redox partners [ferredoxins, NAD(P)H or cytochrome c_3_], that can be used either individually or simultaneously (*via* flavin-based electron bifurcation). [FeFe]-hydrogenases also serve additional physiological functions such as H_2_ uptake (oxidation), H_2_ sensing, and CO_2_ fixation. This broad functional spectrum is enabled by a modular architecture and vast genetic diversity, which is not fully explored and understood. This Mini Review summarises recent advancements in identifying and characterising novel [FeFe]-hydrogenases, which has led to expanding our understanding of their multiple roles in metabolism and functional mechanisms. For example, while numerous well-known [FeFe]-hydrogenases are irreversibly damaged by oxygen, some newly discovered enzymes display intrinsic tolerance. These findings demonstrate that oxygen sensitivity varies between different [FeFe]-hydrogenases: in some cases, protection requires the presence of exogenous compounds such as carbon monoxide or sulphide, while in other cases it is a spontaneous built-in mechanism that relies on a reversible conformational change. Overall, it emerges that additional research is needed to characterise new [FeFe]-hydrogenases as this will reveal further details on the physiology and mechanisms of these enzymes that will enable potential impactful applications.

## Introduction

Microbial hydrogen metabolism is thought to have appeared in the very early days of life on Earth, before oxygen began to accumulate in the atmosphere 2.4 billion years ago (Lyons et al., 2014). It has existed ever since, and it still plays a key role in numerous environments such as soil, wetlands, marine sediments, freshwaters, oceans, geothermal springs, and animal guts (Boyd et al., 2010; Greening et al., 2016; Piche-Choquette and Constant, 2019).

Hydrogenases are specialised metalloenzymes essential to microbial hydrogen metabolism. They are classified in three classes based on the metals found at the active site: [FeFe]-hydrogenases, [NiFe]-hydrogenases, and [Fe]-hydrogenases (also known as Hmd) (Vignais and Billoud, 2007).

[FeFe]-hydrogenases are found in the genome of numerous microorganisms, both Prokaryotes and Eukaryotes but not in Archaea (Peters et al., 2015). Their enzymatic features depend on a biologically unusual iron sulphur centre, named H-cluster, composed of a cubane [4Fe4S]-subcluster linked to a [FeFe]-subcluster *via* a conserved cysteine residue. Concerted proton and electron transfer steps lead to H_2_ production, *via* a mechanism that is under debate (Haumann and Stripp, 2018; Birrell et al., 2021). The ability of [FeFe]-hydrogenases to catalyse reversible H_2_ production at high turnover rates and low overpotential has put them under the spotlight for potential exploitation in devices to produce green hydrogen (Morra et al., 2017; Evans et al., 2019; Brown and King, 2020). [FeFe]-hydrogenases have also inspired the synthesis of artificial catalysts that mimic their natural properties (Ahmed and Dey, 2019; Karayilan et al., 2019). Synthetic biology has also explored the potential of exploiting [FeFe]-hydrogenases *in vivo*, to improve or to instal H_2_ production in several microbial hosts (Klein et al., 2010; Kelly et al., 2015; Noone et al., 2017; Kanygin et al., 2020; Wegelius et al., 2021).

This Mini Review will focus on [FeFe]-hydrogenases and highlight how recent research is revolutionising our understanding of these enzymes.

## [FeFe]-Hydrogenases Diversity: A Poorly Explored Space

**FIGURE 1:**
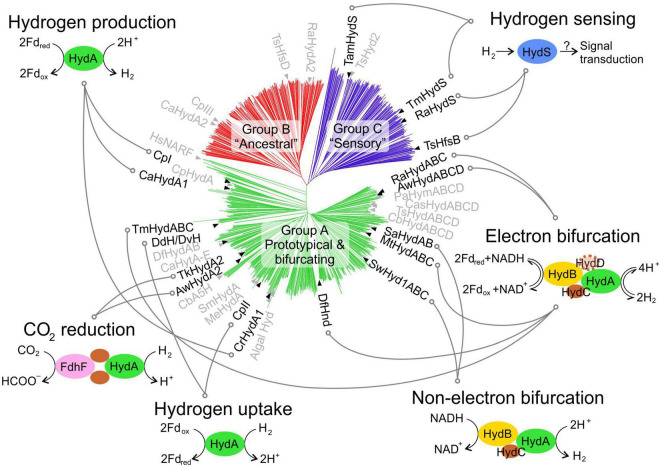
[FeFe]-hydrogenases phylogeny and known functions. A phylogenetic tree shows the phylogeny of [FeFe]-hydrogenase sequences from public databases, as previously proposed (Greening et al., 2016). Enzymes that have been experimentally characterised are indicated on the tree to show their relative position. The proposed physiological function of each enzyme is also presented, where known. A full list of enzymes is available in Supplementary Table 1, including details on the enzyme identifier/acronym used here. Hyd, hydrogenase subunit; FdhF, formate dehydrogenase subunit; Fd_*rex/ox*_, reduced/oxidised ferredoxin; NADH/NAD^+^, reduced/oxidised nicotinamide adenine dinucleotide.

In the post-genomic and multi-omics era, thousands of putative [FeFe]-hydrogenase sequences can be retrieved from public databases. The common factor to all [FeFe]-hydrogenases is the H-domain, a ∼40 kDa (350 amino acids) protein domain that hosts the H-cluster. In addition to sequence variability within this core domain, [FeFe]-hydrogenases display a highly modular genetic organisation, featuring several additional domains and subunits that lead to both monomeric and multi-subunit enzyme complexes. Research in this field has identified massive diversity and several classification schemes have been proposed (Meyer, 2007; Vignais and Billoud, 2007; Calusinska et al., 2010; Winkler et al., 2013). In addition to studying the hydrogenase gene phylogeny, recent studies have also included the analysis of flanking genes. Poudel et al. (2016) compiled 714 sequences and proposed three groups: (G1) monomeric HydA; (G2) trimeric HydABC; (G3) tetrameric HydABCD. Greening et al. (2016) curated 1,222 sequences and proposed a classification into three groups (Figure 1): (A) prototypical and bifurcating; (B) putative ancestral; (C) putative sensory; by analysing variations in the domain organisation and probable quaternary structure, group A can be further split into four subtypes.

However, only three model enzymes have been experimentally characterised to a high level of detail using various techniques, and they all belong to group A (subtype A1). These are: CpI (from the anaerobic nitrogen-fixing bacterium *Clostridium pasteurianum*) (Nakos and Mortenson, 1971; Peters et al., 1998; Therien et al., 2017); DvH (from the sulphate-reducing bacterium *Desulfovibrio vulgaris*) (Legall et al., 1971; Nicolet et al., 1999; Pohorelic et al., 2002); and CrHydA1 (from the eukaryotic green alga *Chlamydomonas reinhardtii*) (Happe and Naber, 1993; Happe and Kaminski, 2002; Mulder et al., 2010). DvH and DdH (from *Desulfovibrio desulfuricans*) sequences have been claimed to be identical (Nicolet et al., 1999), even if a closer inspection at genomes denotes numerous differences. Despite this, the nomenclature has been used interchangeably, and biophysical characterisation has been carried out on DvH sequence under the DdH name (Nicolet et al., 1999; Birrell et al., 2016; Rodriguez-Macia et al., 2020).

From the late 1990s, research has expanded to more [FeFe]-hydrogenases thanks to important technical advances, such as recombinant overexpression (Girbal et al., 2005; King et al., 2006; Kuchenreuther et al., 2010) and production of semi-synthetic hydrogenases (Berggren et al., 2013; Esselborn et al., 2013). Currently, approximately 40 [FeFe]-hydrogenases have been studied experimentally (Figure 1 and Supplementary Table 1). A simple comparison of these numbers with the count of putative [FeFe]-hydrogenases makes it evident that only very little is currently known about these enzymes’ diversity. Furthermore, the level of characterisation is highly variable, and in most cases only a very limited amount of information is available (Supplementary Table 1).

## Beyond Hydrogen Production: Multiple Functional Roles

[FeFe]-hydrogenases are well-known for their prototypical role in hydrogen production (Figure 1): the enzyme acts as a sink for reducing equivalents, allowing for dissipation of excess reducing power from energy metabolism. This role is well-characterised in some clostridial hydrogenases, such as CpI (Therien et al., 2017), and in algal hydrogenases, such as CrHydA1 (Happe and Kaminski, 2002). Other hydrogenases with very high sequence similarity are believed to acts in a similar way both within green algae (Florin et al., 2001; Winkler et al., 2002; Kamp et al., 2008; Meuser et al., 2011; Cornish et al., 2015) and Clostridia (Demuez et al., 2007; Morra et al., 2016b). However, H_2_-producing hydrogenases do not cluster together in the phylogenetic tree, demonstrating that the function cannot simply be predicted from the sequence alone, as previously noted (Greening et al., 2016).

In addition to this flagship role, several new functions have been identified. During hydrogen uptake (Figure 1), the enzyme oxidises hydrogen and transfers the low potential electrons to a suitable cellular acceptor. This function has been proposed for relatively few [FeFe]-hydrogenases, such as the cytoplasmic CpII (Therien et al., 2017) and periplasmic DdH/DvH (Pohorelic et al., 2002).

Reactions of additional complexity are catalysed by multi-subunit [FeFe]-hydrogenases. AwHydA2 (from *Acetobacterium woodii*) (Schuchmann and Muller, 2013) and TkHydA2 (from *Thermoanaerobacter kivui*) (Schwarz et al., 2018) have been shown to form stable heterotetrameric complexes with two FeS subunits and a formate dehydrogenase (FdhF) subunit. These enzymes have been named HDCR (Hydrogen-Dependent Carbon dioxide Reductase) after their unprecedented ability to catalyse direct CO_2_ reduction using H_2_ as the sole reducing agent, in a single and efficient step (Schuchmann et al., 2018; Leo et al., 2021).

Following the discovery of TmHydABC (from *Thermotoga maritima*) (Schut and Adams, 2009), numerous other heterotrimeric and heterotetrameric [FeFe]-hydrogenases have been shown to perform flavin-based electron bifurcation (FBEB). These enzymes couple the thermodynamically favourable oxidation of ferredoxin to the unfavourable oxidation of NADH leading to H_2_ production. The coupling is synergistic and provides a clear physiological advantage over “conventional” hydrogen production from ferredoxin only, as it allows simultaneous reoxidation of both NADH and ferredoxin from glycolysis, thus facilitating ATP production in the absence of aerobic respiration (Buckel and Thauer, 2018; Peters et al., 2018; Schuchmann et al., 2018).

Two [FeFe]-hydrogenases SwHyd1ABC (from *Syntrophomonas wolfei*) (Losey et al., 2017) and SaHydAB (from *Syntrophus aciditrophicus*) (Losey et al., 2020) have been discovered to catalyse H_2_ production from NADH without the requirement of reduced ferredoxin. Despite overall sequence and predicted structural similarity to FBEB enzymes, closer inspection of the flavin-containing HydB subunit revealed differences that may explain the absence of synergistic bifurcation (Losey et al., 2020).

A sensory role has been proposed for a number of group C [FeFe]-hydrogenases, such as TmHydS (Chongdar et al., 2018) and TamHydS (Land et al., 2020). This function has been proposed based the presence of a PAS domain, that is known to take part in signal transduction of other proteins. Furthermore, the lack of a conserved cysteine near the H-cluster slows down the turnover rate, thus making unlikely an active metabolic role. Additional evidence supporting a sensory role has been reported for RaHydS (from *Ruminococcus albus*) (Zheng et al., 2014) and TsHfsB (from *Thermoanaerobacterium saccharolyticum*) (Shaw et al., 2009). However, given the little direct physiological evidence for such role, this assignment is considered putative.

Group B is currently much less characterised and a tentative denomination as “ancestral” has been proposed, but due to lack of evidence it is difficult to discuss further on their function. Some data are available for CpIII, showing that the gene is transcribed (Therien et al., 2017) and that the enzyme is active and displays a different catalytic bias to both CpI and CpII (Artz et al., 2020).

It is also notable that proteins with sequence similarity to [FeFe]-hydrogenases can be found in higher eukaryotes, including humans (HsNARF, Figure 1), where they lost H_2_-linked functionality and evolved to other roles (Barton and Worman, 1999; Ding et al., 2020).

The impressive variety of functional roles for [FeFe]-hydrogenases is not surprising given the large number of organisms that rely on them, and their evolutionary history that allowed adaptation to numerous ecological niches. However, such diversity does not only occur across different organisms, but also within them: several species possess multiple [FeFe]-hydrogenase genes annotated in the genome, often in addition to other H_2_-activating enzymes such as [NiFe]-hydrogenases and nitrogenases (Calusinska et al., 2010; Baffert et al., 2019). The apparent redundancy of hydrogen-related enzymes may provide advantages by quickly adapting the metabolism in response to the environmental changes. However, a comprehensive investigation on multiple hydrogenases within an organism is currently missing.

*Thermoanaerobacterium saccharolyticum* has four putative [FeFe]-hydrogenases and a putative [NiFe]-hydrogenase genes. A systematic knockout study revealed that the *hfs* genes encoding for TsHfsB and TsHfsD [FeFe]-hydrogenases are essential for H_2_ production, while deletion of the other genes had no effect on this function. Moreover, deletion of *hfs* genes downregulated the expression of all the other genes (*hyd* and *ech*), suggesting a regulatory or sensory role for TsHfsB. Consequently, group B TsHfsD may be the main enzyme for H_2_ production in this organism (Shaw et al., 2009).

Within green algae, it is common to find two closely related [FeFe]-hydrogenase genes that are likely originating from gene duplication (Meuser et al., 2011). For example, *Chlamydomonas reinhardtii* encodes for CrHydA1 and CrHydA2, whose expression profile is very similar (Forestier et al., 2003). The two isoenzymes differ for their affinity for the ferredoxin PetF and for catalytic bias, suggesting different functional roles (Engelbrecht et al., 2021).

The well-known solvent producer *Clostridium acetobutylicum* has two [FeFe]-hydrogenases (CaHydA1 and CaHydA2), a [NiFe]-hydrogenase and nitrogenase. It has been suggested that CaHydA1 is the main enzyme for hydrogen production during acidogenesis (Demuez et al., 2007), but more recently a ΔCaHydA1 mutant strain has shown abundant H_2_ production (Du et al., 2021) suggesting a significant contribution from other enzymes. On the other hand, the [NiFe]-hydrogenase has been shown to play a key role in hydrogen cycling during solventogenesis (i.e., H_2_ reoxidation to provide reducing power for the acid-to-solvent conversion) (Germane et al., 2018).

In the case of *Clostridium pasteurianum* (three [FeFe]-hydrogenases, a [NiFe]-hydrogenase and nitrogenase), it has been proposed that CpI is the key H_2_ producer under non-nitrogen-fixing conditions (Adams, 1990), while CpII and the [NiFe]-hydrogenase would act as H_2_ uptake enzymes during nitrogen fixation. This would allow for reducing equivalents to be recovered from the highly uncoupled reaction of nitrogenase, ultimately improving the efficiency of this energy-consuming process (Therien et al., 2017).

Gene expression studies of multiple [FeFe]-hydrogenases in *Clostridium butyricum*, *Clostridium beijerinckii*, and Clostridium *perfringens* have shown that all genes are transcribed and regulation is actively occurring, suggesting different functional roles that have not yet been determined (Morra et al., 2014; Calusinska et al., 2015; Arizzi et al., 2021).

## Oxygen Sensitivity: Not an Insurmountable Problem

**FIGURE 2:**
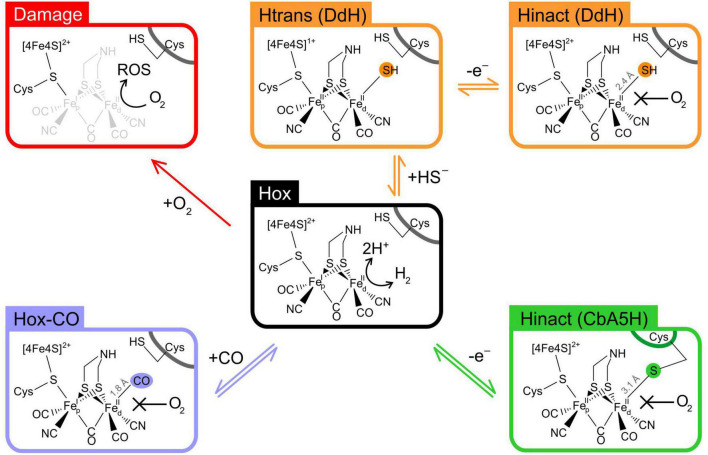
Oxygen tolerance strategies in [FeFe]-hydrogenases. Schematic representation of the H-cluster in the oxidised active state Hox (centre). In the absence of any exogenous protectant, numerous [FeFe]-hydrogenases undergo irreversible inactivation due to H-cluster damage with loss of Fe atoms (red pathway); carbon monoxide acts as a protective agent due to its ability to form Hox-CO, by binding reversibly to the H-cluster at the same site as O_2_ (purple pathway); in DdH, a similar mechanism occurs when sulphide binds to the H-cluster forming Hinact, *via* the Htrans intermediate (orange pathway); in CbA5H, a conformational change in the protein structure allows for a conserved cysteine to directly bind to the H-cluster, forming Hinact (green pathway). Fe_*p*_, proximal iron atom; Fe_*d*_, distal iron atom; Cys, cysteine residue.

For long time it has been assumed that [FeFe]-hydrogenases were extremely sensitive to O_2_, with their catalytic activity disappearing irreversibly and very quickly, in contrast to [NiFe]-hydrogenases that often display reversible inhibition or complete tolerance (Goldet et al., 2009; Lautier et al., 2011; Kubas et al., 2017). While this is certainly true for some of the most studied model [FeFe]-hydrogenases, the characterisation of novel enzymes has recently revealed that the phenomenon of oxygen sensitivity is highly variable across the class, and several examples of oxygen-stable enzymes exist (Figure 2).

Studies on model enzymes CrHydA1 and CpI have shown that the H-cluster suffers severe and irreversible structural damage when exposed to oxygen, resulting in loss of several Fe atoms and their non-protein ligands (Stripp et al., 2009; Swanson et al., 2015; Esselborn et al., 2019). The exact degradation mechanism is still under debate, but there is agreement that this requires O_2_ binding to the distal iron atom (Fe_*d*_) followed by electron and proton transfer, leading to the formation of reactive oxygen species (ROS) that in turn would cause the actual damage (Figure 2). Protection from oxygen can occur in the presence of carbon monoxide *via* competition, as CO is able to bind to Fe_*d*_ faster than oxygen, forming Hox-CO (Lemon and Peters, 1999; Goldet et al., 2009).

Oxygen sensitivity of [FeFe]-hydrogenases is an obvious practical limitation to working with such enzymes and raises concerns over their prospective exploitation for potential applications (Ghirardi, 2015; Karayilan et al., 2019). As such, several attempts have been made to improve oxygen tolerance of model [FeFe]-hydrogenases, adopting both rational and random mutagenesis approaches but the improvements reported are limited (Lautier et al., 2011; Bingham et al., 2012; King et al., 2014).

More progress has been made by looking at other [FeFe]-hydrogenases. For example, it has been known for decades that DdH and DvH can be purified under air as an inactive state (named Hinact) that can be reactivated by a reductive treatment (Pierik et al., 1998; Roseboom et al., 2006). However, the first attempts to generate Hinact *in vitro* were unsuccessful, and the exact protection mechanism has been elusive for years. Only recently it was shown that DdH requires the addition of exogenous sulphide to form Hinact, *via* the intermediate species Htrans. It has been demonstrated that Hinact in DdH (Figure 2) is an overoxidized H-cluster with sulphide bound to Fe_*d*_, thus preventing O_2_ binding by direct competition and protecting the enzyme from damage. Given the importance of sulphur metabolism in *Desulfovibrio*, it has been suggested that this mechanism may play a physiological role *in vivo*. Interestingly, sulphide dependent Hinact formation is not an exclusive feature of DdH, as it occurs in CrHydA1 as well; however, this mechanism is not effective on CpI, highlighting variability between different [FeFe]-hydrogenases (Rodriguez-Macia et al., 2018; Rodriguez-Macia et al., 2020).

Also recently, it has been shown that CbA5H from *C. beijerinckii* is able to form Hinact in a fully reversible manner; the enzyme can be inactivated and reactivated multiple times without any loss of activity (Morra et al., 2016a). Further characterisation showed that Hinact formation in CbA5H does not require exogenous sulphide and spectro-electrochemical titrations showed that the Hox/Hinact transition in CbA5H is a 1-electron process that occurs at an unusually low potential, without forming Htrans (Corrigan et al., 2020). Xray crystallography has recently confirmed that Hinact in CbA5H is indeed independent of exogenous sulphide, as a sulphur atom from a conserved cysteine can bind directly Fe_*d*_, following a conformational change within the enzyme (Figure 2). This is particularly fascinating since the immediate surroundings of the H-cluster in CbA5H are conserved and do not differ from other [FeFe]-hydrogenases. Hence, it has been proposed that the protection mechanism depends on three non-conserved amino acids, situated far away from the H-cluster, that allow for the local conformational change to occur. This finding highlights the complexity of the interplay between the H-cluster and the protein residues as a fundamental part of [FeFe]-hydrogenase function, challenging the common assumption of [FeFe]-hydrogenases being a rigid scaffold that simply hosts a metal cluster responsible for their peculiar features (Winkler et al., 2021).

Protection from oxygen is not limited to few fortunate cases, as it has been shown that two other [FeFe]-hydrogenases are likely to display similar protection mechanisms: DfHnd, the heterotetrameric bifurcating [FeFe]-hydrogenase from *Desulfovibrio fructosovorans* forms Hinact and is protected from oxygen damage (Kpebe et al., 2018); also CpIII can form a species whose spectroscopy is reminiscent of Hinact (Artz et al., 2020).

Overall, recent research has highlighted that oxygen sensitivity/tolerance in [FeFe]-hydrogenases is much more complex than previously assumed. Different enzymes display completely different reactivity toward oxygen, with a growing number of them being able to tolerate it. It emerges that long term protection from oxygen damage in [FeFe]-hydrogenases invariably requires a nucleophilic species (CO, H_2_S or a cysteine thiol) to directly bind Fe_*d*_ thus competing for O_2_ binding (Figure 2). While carbon monoxide protection seems to be a universal feature of all hydrogenases, sulphur-based protection appears to vary significantly between different enzymes. This clearly highlights that the protein environment plays a crucial role in determining the enzyme’s fate when exposed to O_2_, but the exact determinants for such a diversity are not comprehensively understood. Also, the physiological implications of oxygen tolerance in [FeFe]-hydrogenases have not been fully addressed yet.

## Conclusion and Future Perspectives

Recent characterisation of novel [FeFe]-hydrogenases has highlighted a broad functional spectrum that exceeds their prototypical role in hydrogen production. Involvement in hydrogen sensing, electron bifurcation and CO_2_ reduction demonstrate how diverse [FeFe]-hydrogenase functions are, and how crucial they are to support cellular metabolism under different conditions. It has also become clear that several [FeFe]-hydrogenases have evolved strategies to cope with oxygen, making them oxygen tolerant.

Despite the major advancements summarised here, the current understanding of [FeFe]-hydrogenase diversity is still limited because few enzymes have been characterised in detail so far. Studying novel [FeFe]-hydrogenases and populating the phylogenetic tree with experimental evidence from this unexplored space will be essential to improve our understanding of [FeFe]-hydrogenase function.

Research in this field has shown that relying mainly on few model enzymes and extrapolating information, in the assumption that they represent a vast enzyme class, inevitably leads to severe biases. Providing accurate predictions purely based on primary sequence data and phylogenetic positioning will always be a risky task but it is reasonable to expect that expanding our knowledge to additional enzymes will make comparisons between closely related sequences more reliable. Improving our understanding of [FeFe]-hydrogenase function will provide numerous benefits, for example when interpreting-omics data, as this task heavily relies on previous information being available and correctly annotated in databases. Also, the availability of additional enzymes will inevitably expand the portfolio of [FeFe]-hydrogenases with desirable features for a given application, either *in vitro* in a technological device, or *in vivo* within an engineered organism, or as an inspirational example for artificial catalysts, thus increasing the chances for success in future applications.

## Author Contributions

The author confirms being the sole contributor of this work and has approved it for publication.

## Conflict of Interest

The author declares that the research was conducted in the absence of any commercial or financial relationships that could be construed as a potential conflict of interest.

## Publisher’s Note

All claims expressed in this article are solely those of the authors and do not necessarily represent those of their affiliated organizations, or those of the publisher, the editors and the reviewers. Any product that may be evaluated in this article, or claim that may be made by its manufacturer, is not guaranteed or endorsed by the publisher.
