# Supplementary material for: Fantastic [FeFe]-Hydrogenases and Where to Find Them
Source: Front Microbiol. 2022 Mar 2;13:853626. doi: 10.3389/fmicb.2022.853626 (PMC8924675; doi:10.3389/fmicb.2022.853626)
Supplement: Supplementary file 1 [file Table_1.DOCX]

Fantastic [FeFe]-hydrogenases and where to find them

Simone Morra

Supplementary Material

# Supplementary Table 1

List of all [FeFe]-hydrogenases whose characterization has been reported as of December 2021. The same acronym/identifier has been used in Figure 1 of the main text. The identifier begins with two letters that represent the organism source, followed by an acronym that refers to the specific enzyme (either a monomer or a protein complex). Wherever possible, it has been chosen to match previous literature. The group classification is according to (Greening et al., 2016). The type of characterization available is simplified for consistency and clarity, additional information can be found in the cited literature. In some cases, the cited literature is necessarily a selection, and it is not intended to be exhaustive.

| **Organism** | **Acronym/ identifier** | **Group** | **NCBI sequence ID** | **First report year** | **Characterization available, reference(s)** |
| --- | --- | --- | --- | --- | --- |
| *Acetobacterium woodii* | AwHydABCD | A3 | WP014357048.1 | 2012 | Purification (native) (Schuchmann and Muller, 2012) |
|  | AwHydA2 (HDCR) | A4 | WP014355220.1 | 2013 | Purification (native) (Schuchmann and Muller, 2013)  Purification (recombinant) (Leo et al., 2021) |
| *Caldanaerobacter subterraneus* | CasHydABCD | A3 | WP009609852.1 | 2004 | Purification (native) (Soboh et al., 2004)  Used for synthetic biology (Kelly et al., 2015) |
| *Caldicellulosiruptor bescii* | CbHydABCD | A3 | WP015907776.1 | 2016 | Purification (Poudel et al., 2016) |
| *Chlamydomonas moewusii* | CmHydA1 | A1 | n/a | 2008 | Purification (native), spectroscopy (Kamp et al., 2008)  Transcriptomics (Yang et al., 2013) |
| *Chlamydomonas reinhardtii* | CrHydA1 | A1 | XP001693376.1 | 1993 | Purification (native) (Happe and Naber, 1993)  Purification (recombinant) (Girbal et al., 2005;King et al., 2006;Sybirna et al., 2008;von Abendroth et al., 2008;Kuchenreuther et al., 2010)  Purification (semi-synthetic) (Berggren et al., 2013;Esselborn et al., 2013)  Expression regulation (Happe and Kaminski, 2002)  X-ray crystallography (Mulder et al., 2010;Swanson et al., 2015)  Spectroscopy, mutagenesis, electrochemistry (Goldet et al., 2009;Silakov et al., 2009;Mulder et al., 2013;Mulder et al., 2014;Senger et al., 2016;Kertess et al., 2017;Lampret et al., 2017;Meszaros et al., 2020) |
|  | CrHydA2 | A1 | XP001694503.1 | 2003 | Expression regulation (Forestier et al., 2003)  Purification (recombinant) (King et al., 2006)  Purification (semi-synthetic) (Engelbrecht et al., 2021) |
| *Chlorella fusca* | CfHydA | A1 | CAC83290.1 | 2002 | Purification (native) (Winkler et al., 2002) |
| *Chlorella variabilis* | CvHydA1 | A1 | XP005848612.1 | 2011 | Expression regulation, *in vivo* function (Meuser et al., 2011)  Purification (semi-synthetic), (Engelbrecht et al., 2017) |
| *Chlorococcum submarinum* | CsHydA | A1 | n/a | 2008 | Purification (native), spectroscopy (Kamp et al., 2008) |
| *Clostridium acetobutylicum* | CaHydA1 | A1 | WP010963357.1 | 1996 | Gene identification, expression regulation  (Gorwa et al., 1996)  Purification (recombinant) (Girbal et al., 2005;King et al., 2006;von Abendroth et al., 2008;Morra et al., 2015)  Physiology and metabolism (Demuez et al., 2007;Klein et al., 2010;Cooksley et al., 2012;Du et al., 2021)  Spectroscopy, mutagenesis, electrochemistry (Goldet et al., 2009;Lautier et al., 2011;Morra et al., 2016b;Ratzloff et al., 2018) |
|  | CaHydA2 (CaHydB) | B | WP010966505.1 | 2006 | Purification (recombinant) (King et al., 2006) |
| *Clostridium autoethanogenum* | CaHytA-E/FdhA | A4 | WP023162857.1 | 2013 | Purification (native) (Wang et al., 2013a) |
| *Clostridium beijerinckii* | CbA5H (Cbei_1773) | A1 | WP026887313.1 | 2016 | Purification (recombinant), spectroscopy (Morra et al., 2016a)  Purification (semi-synthetic), spectroscopy, electrochemistry (Corrigan et al., 2020)  X-ray crystallography, mutagenesis, electrochemistry (Winkler et al., 2021) |
|  | Cbei_1773, Cbei_4110, Cbei_0327, Cbei_1901, Cbei_4000, Cbei_3796 | n/a | n/a | 2014 | Expression regulation (Morra et al., 2014;Arizzi et al., 2021) |
| *Clostridium butyricum* | HydA2, HydA8, HydB2, HydB3 | n/a | n/a | 2015 | Expression regulation (Calusinska et al., 2015) |
| *Clostridium pasteurianum* | CpI | A1 | WP004455619.1 | 1971 | Purification (native) (Nakos and Mortenson, 1971)  Purification (recombinant) (Kuchenreuther et al., 2010)  Purification (semi-synthetic) (Esselborn et al., 2013)  X-ray crystallography (Peters et al., 1998;Lemon and Peters, 1999;Esselborn et al., 2016;Duan et al., 2018;Esselborn et al., 2019;Artz et al., 2020)  Spectroscopy, mutagenesis, electrochemistry (Chen et al., 2002;Cornish et al., 2011;Bingham et al., 2012;Winkler et al., 2017;Artz et al., 2020;Morra et al., 2021) |
|  | CpII | A | WP003446424.1 | 1978 | Purification (native) (Chen and Blanchard, 1978)  Spectroscopy (Adams and Mortenson, 1984)  Expression regulation (Therien et al., 2017)  Purification (recombinant), spectroscopy, electrochemistry (Artz et al., 2020) |
|  | CpIII | B | WP003447632.1 | 2017 | Expression regulation (Therien et al., 2017)  Purification (recombinant), spectroscopy, electrochemistry (Artz et al., 2020) |
| *Clostridium perfringens* | CpHydA | A1 | WP011593109.1 | 1999 | Expression regulation (Kaji et al., 1999)  Purification (recombinant), spectroscopy (Morra et al., 2016c) |
| *Desulfovibrio fructosovorans* | DfHnd | A1 | WP005996594.1 | 1995 | Gene identification, enzyme activity (Malki et al., 1995)  Purification (native), spectroscopy (Kpebe et al., 2018) |
|  | DfHydAB | A3 | WP005990603.1 | 1998 | Purification (native) (Casalot et al., 1998)  Gene knockout (Casalot et al., 2002) |
| *Desulfovibrio vulgaris* Hildenborough | DvH (DdH) | A1 | WP010939057.1 | 1971 | Gene identification, purification (native) (Legall et al., 1971;Voordouw and Brenner, 1985)  Gene knockout (Pohorelic et al., 2002)  Spectroscopy, electrochemistry (Vincent et al., 2005;Albracht et al., 2006;Roseboom et al., 2006;Goldet et al., 2009;Birrell et al., 2016;Rodriguez-Macia et al., 2017;Rodriguez-Macia et al., 2018)  X-ray crystallography, spectroscopy (Nicolet et al., 1999;Nicolet et al., 2001;Rodriguez-Macia et al., 2020) |
| *Megasphaera elsdenii* | MeHydA | A1 | WP014016432.1 | 1979 | Purification (native) (Vandijk et al., 1979)  Purification (semi-synthetic), spectroscopy, protein engineering (Esselborn et al., 2013;Caserta et al., 2016;Caserta et al., 2018) |
| *Moorella thermoacetica* | MtHydABC | A3 | WP011393219.1 | 2013 | Purification (native) (Wang et al., 2013b) |
| *Peptoclostridium acidaminophilum* | PaHymABCD | A | CAC39231.1 | 2003 | Expression regulation (Graentzdoerffer et al., 2003) |
| *Ruminococcus albus* | RaHydABC, RaHydA2, RaHydS | A3, B, C | WP002853139.1, WP002848990.1, WP002849014.1 | 2014 | Expression regulation, purification (native) (Zheng et al., 2014) |
| *Solobacterium moorei* | SmHydA | A1 | WP028077982.1 | 2019 | Purification (semi-synthetic) (Land et al., 2019) |
| *Syntrophomonas wolfei* | SwHyd1ABC | A3 | WP011640436.1 | 2017 | Purification (recombinant) (Losey et al., 2017) |
| *Syntrophus aciditrophicus* | SaHydAB | A3 | WP011417004.1 | 2020 | Purification (recombinant) (Losey et al., 2020) |
| *Thermoanaerobacter kivui* | TkHydA2  (HDCR) | A4 | AIS53140.1 | 2018 | Purification (native) (Schwarz et al., 2018) |
| *Thermoanaerobacter mathranii* | TamHydS | C | WP013150113.1 | 2019 | Purification (semi-synthetic) (Land et al., 2019;Land et al., 2020) |
| *Thermoanaerobacterium saccharolyticum* | TsHydABCD/ Hyd2/HfsB/HfsD | C, C, B, A | WP014758976.1, WP014758428.1, ACU11597.1, ACA51661.1 | 2009 | Expression regulation, gene knockout (Shaw et al., 2009) |
| *Thermotoga maritima* | TmHydS | C | WP004081666.1 | 2018 | Purification (semi-synthetic), spectroscopy (Chongdar et al., 2018) |
|  | TmHydABC | A3 | WP004081677.1 | 1999 | Purification (native) (Verhagen et al., 1999;Schut and Adams, 2009)  Purification (semi-synthetic), spectroscopy, electrochemistry (Chongdar et al., 2020)  X-ray crystallography (Furlan et al., 2021) |
| *Tetradesmus obliquus* | ToHydA | A1 | CAC34419.1 | 2001 | Expression regulation, purification (native) (Florin et al., 2001) |
| *Volvox carteri* | VcHydA1, VcHydA2 | A1, A1 | XP002948487.1, XP002948483.1 | 2015 | Expression regulation, purification (recombinant) (Cornish et al., 2015) |

**Supplementary references.**

Adams, M.W.W., and Mortenson, L.E. (1984). THE PURIFICATION OF HYDROGENASE-II (UPTAKE HYDROGENASE) FROM THE ANAEROBIC N-2-FIXING BACTERIUM CLOSTRIDIUM-PASTEURIANUM. *Biochimica Et Biophysica Acta* 766**,** 51-61.

Albracht, S.P.J., Roseboom, W., and Hatchikian, E.C. (2006). The active site of the [FeFe]-hydrogenase from Desulfovibrio desulfuricans. I. Light sensitivity and magnetic hyperfine interactions as observed by electron paramagnetic resonance. *JBIC Journal of Biological Inorganic Chemistry* 11**,** 88-101.

Arizzi, M., Morra, S., Gilardi, G., Pugliese, M., Gullino, M.L., and Valetti, F. (2021). Improving sustainable hydrogen production from green waste: FeFe -hydrogenases quantitative gene expression RT-qPCR analysis in presence of autochthonous consortia. *Biotechnology for Biofuels* 14**,** 17.

Artz, J.H., Zadvornyy, O.A., Mulder, D.W., Keable, S.M., Cohen, A.E., Ratzloff, M.W., Williams, S.G., Ginovska, B., Kumar, N., Song, J.H., Mcphillips, S.E., Davidson, C.M., Lyubimov, A.Y., Pence, N., Schut, G.J., Jones, A.K., Soltis, S.M., Adams, M.W.W., Raugei, S., King, P.W., and Peters, J.W. (2020). Tuning Catalytic Bias of Hydrogen Gas Producing Hydrogenases. *Journal of the American Chemical Society* 142**,** 1227-1235.

Berggren, G., Adamska, A., Lambertz, C., Simmons, T.R., Esselborn, J., Atta, M., Gambarelli, S., Mouesca, J.M., Reijerse, E., Lubitz, W., Happe, T., Artero, V., and Fontecave, M. (2013). Biomimetic assembly and activation of FeFe -hydrogenases. *Nature* 499**,** 66-+.

Bingham, A.S., Smith, P.R., and Swartz, J.R. (2012). Evolution of an FeFe hydrogenase with decreased oxygen sensitivity. *International Journal of Hydrogen Energy* 37**,** 2965-2976.

Birrell, J.A., Wrede, K., Pawlak, K., Rodriguez-Macia, P., Rudiger, O., Reijerse, E.J., and Lubitz, W. (2016). Artificial Maturation of the Highly Active Heterodimeric FeFe Hydrogenase from Desulfovibrio desulfuricans ATCC 7757. *Israel Journal of Chemistry* 56**,** 852-863.

Calusinska, M., Hamilton, C., Monsieurs, P., Mathy, G., Leys, N., Franck, F., Joris, B., Thonart, P., Hiligsmann, S., and Wilmotte, A. (2015). Genome-wide transcriptional analysis suggests hydrogenase- and nitrogenase-mediated hydrogen production in Clostridium butyricum CWBI 1009. *Biotechnology for Biofuels* 8.

Casalot, L., Hatchikian, C.E., Forget, N., De Philip, P., Dermoun, Z., Belaich, J.P., and Rousset, M. (1998). Molecular study and partial characterization of iron-only hydrogenase in Desulfovibrio fructosovorans. *Anaerobe* 4**,** 45-55.

Casalot, L., Valette, O., De Luca, G., Dermoun, Z., Rousset, M., and De Philip, P. (2002). Construction and physiological studies of hydrogenase depleted mutants of Desulfovibrio fructosovorans. *Fems Microbiology Letters* 214**,** 107-112.

Caserta, G., Adamska-Venkatesh, A., Pecqueur, L., Atta, M., Artero, V., Roy, S., Reijerse, E., Lubitz, W., and Fontecave, M. (2016). Chemical assembly of multiple metal cofactors: The heterologously expressed multidomain FeFe -hydrogenase from Megasphaera elsdenii. *Biochimica Et Biophysica Acta-Bioenergetics* 1857**,** 1734-1740.

Caserta, G., Papini, C., Adamska-Venkatesh, A., Pecqueur, L., Sommer, C., Reijerse, E., Lubitz, W., Gauquelin, C., Meynial-Salles, I., Pramanik, D., Artero, V., Atta, M., Del Barrio, M., Faivre, B., Fourmond, V., Leger, C., and Fontecave, M. (2018). Engineering an FeFe -Hydrogenase: Do Accessory Clusters Influence O-2 Resistance and Catalytic Bias? *Journal of the American Chemical Society* 140**,** 5516-5526.

Chen, J.S., and Blanchard, D.K. (1978). ISOLATION AND PROPERTIES OF A UNIDIRECTIONAL H-2-OXIDIZING HYDROGENASE FROM STRICTLY ANAEROBIC N-2-FIXING BACTERIUM CLOSTRIDIUM-PASTEURIANUM-W5. *Biochemical and Biophysical Research Communications* 84**,** 1144-1150.

Chen, Z.J., Lemon, B.J., Huang, S., Swartz, D.J., Peters, J.W., and Bagley, K.A. (2002). Infrared studies of the CO-inhibited form of the Fe-only hydrogenase from Clostridium pasteurianum I: Examination of its light sensitivity at cryogenic temperatures. *Biochemistry* 41**,** 2036-2043.

Chongdar, N., Birrell, J.A., Pawlak, K., Sommer, C., Reijerse, E.J., Rudiger, O., Lubitz, W., and Ogata, H. (2018). Unique Spectroscopic Properties of the H-Cluster in a Putative Sensory FeFe Hydrogenase. *Journal of the American Chemical Society* 140**,** 1057-1068.

Chongdar, N., Pawlak, K., R?Diger, O., Reijerse, E.J., Rodr?Guez-Maci, P., Lubitz, W., Birrell, J.A., and Ogata, H. (2020). Spectroscopic and biochemical insight into an electron-bifurcating FeFe hydrogenase. *Journal of Biological Inorganic Chemistry* 25**,** 135-149.

Cooksley, C.M., Zhang, Y., Wang, H.Z., Redl, S., Winzer, K., and Minton, N.P. (2012). Targeted mutagenesis of the Clostridium acetobutylicum acetone-butanol-ethanol fermentation pathway. *Metabolic Engineering* 14**,** 630-641.

Cornish, A.J., Gartner, K., Yang, H., Peters, J.W., and Hegg, E.L. (2011). Mechanism of Proton Transfer in FeFe -Hydrogenase from Clostridium pasteurianum. *Journal of Biological Chemistry* 286**,** 38341-38347.

Cornish, A.J., Green, R., Gartner, K., Mason, S., and Hegg, E.L. (2015). Characterization of Hydrogen Metabolism in the Multicellular Green Alga Volvox carteri. *Plos One* 10**,** 15.

Corrigan, P.S., Tirsch, J.L., and Silakov, A. (2020). Investigation of the Unusual Ability of the FeFe Hydrogenase from Clostridium beijerinckii to Access an O-2-Protected State. *Journal of the American Chemical Society* 142**,** 12409-12419.

Demuez, M., Cournac, L., Guerrini, O., Soucaille, P., and Girbal, L. (2007). Complete activity profile of Clostridium acetobutylicum FeFe -hydrogenase and kinetic parameters for endogenous redox partners. *Fems Microbiology Letters* 275**,** 113-121.

Du, G.Q., Che, J., Wu, Y.D., Wang, Z.Z., Jiang, Z.Y., Ji, F., and Xue, C. (2021). Disruption of hydrogenase gene for enhancing butanol selectivity and production in Clostridium acetobutylicum. *Biochemical Engineering Journal* 171**,** 5.

Duan, J.F., Senger, M., Esselborn, J., Engelbrecht, V., Wittkamp, F., Apfel, U.P., Hofmann, E., Stripp, S.T., Happe, T., and Winkler, M. (2018). Crystallographic and spectroscopic assignment of the proton transfer pathway in FeFe -hydrogenases. *Nature Communications* 9**,** 11.

Engelbrecht, V., Liedtke, K., Rutz, A., Yadav, S., Gunzel, A., and Happe, T. (2021). One isoform for one task? The second hydrogenase of Chlamydomonas reinhardtii prefers hydrogen uptake. *International Journal of Hydrogen Energy* 46**,** 7165-7175.

Engelbrecht, V., Rodriguez-Macia, P., Esselborn, J., Sawyer, A., Hemschemeier, A., Rudiger, O., Lubitz, W., Winkler, M., and Happe, T. (2017). The structurally unique photosynthetic Chlorella variabilis NC64A hydrogenase does not interact with plant-type ferredoxins. *Biochimica Et Biophysica Acta-Bioenergetics* 1858**,** 771-778.

Esselborn, J., Kertess, L., Apfel, U.P., Hofmann, E., and Happe, T. (2019). Loss of Specific Active-Site Iron Atoms in Oxygen-Exposed FeFe -Hydrogenase Determined by Detailed X-ray Structure Analyses. *Journal of the American Chemical Society* 141**,** 17721-17728.

Esselborn, J., Lambertz, C., Adamska-Venkatesh, A., Simmons, T., Berggren, G., Nothl, J., Siebel, J., Hemschemeier, A., Artero, V., Reijerse, E., Fontecave, M., Lubitz, W., and Happe, T. (2013). Spontaneous activation of FeFe -hydrogenases by an inorganic 2Fe active site mimic. *Nature Chemical Biology* 9**,** 607-609.

Esselborn, J., Muraki, N., Klein, K., Engelbrecht, V., Metzler-Nolte, N., Apfel, U.P., Hofmann, E., Kurisu, G., and Happe, T. (2016). A structural view of synthetic cofactor integration into FeFe -hydrogenases. *Chemical Science* 7**,** 959-968.

Florin, L., Tsokoglou, A., and Happe, T. (2001). A novel type of iron hydrogenase in the green alga Scenedesmus obliquus is linked to the photosynthetic electron transport chain. *Journal of Biological Chemistry* 276**,** 6125-6132.

Forestier, M., King, P., Zhang, L.P., Posewitz, M., Schwarzer, S., Happe, T., Ghirardi, M.L., and Seibert, M. (2003). Expression of two Fe -hydrogenases in Chlamydomonas reinhardtii under anaerobic conditions. *European Journal of Biochemistry* 270**,** 2750-2758.

Furlan, C., Chongdar, N., Gupta, P., Lubitz, W., Ogata, H., Blaza, J., and Birrell, J. (2021). "Structural insight on the mechanism of an electron-bifurcating [FeFe] hydrogenase". (*ChemRxiv* . doi:10.33774/chemrxiv-2021-m2jgl. This content is a preprint and has not been peer-reviewed.).

Girbal, L., Von Abendroth, G., Winkler, M., Benton, P.M.C., Meynial-Salles, I., Croux, C., Peters, J.W., Happe, T., and Soucaille, P. (2005). Homologous and heterologous overexpression in Clostridium acetobutylicum and characterization of purified clostridial and algal Fe-only hydrogenases with high specific activities. *Applied and Environmental Microbiology* 71**,** 2777-2781.

Goldet, G., Brandmayr, C., Stripp, S.T., Happe, T., Cavazza, C., Fontecilla-Camps, J.C., and Armstrong, F.A. (2009). Electrochemical Kinetic Investigations of the Reactions of FeFe -Hydrogenases with Carbon Monoxide and Oxygen: Comparing the Importance of Gas Tunnels and Active-Site Electronic/Redox Effects. *Journal of the American Chemical Society* 131**,** 14979-14989.

Gorwa, M.F., Croux, C., and Soucaille, P. (1996). Molecular characterization and transcriptional analysis of the putative hydrogenase gene of Clostridium acetobutylicum ATCC 824. *Journal of Bacteriology* 178**,** 2668-2675.

Graentzdoerffer, A., Rauh, D., Pich, A., and Andreesen, J.R. (2003). Molecular and biochemical characterization of two tungsten- and selenium-containing formate dehydrogenases from Eubacterium acidaminophilum that are associated with components of an iron-only hydrogenase. *Archives of Microbiology* 179**,** 116-130.

Greening, C., Biswas, A., Carere, C.R., Jackson, C.J., Taylor, M.C., Stott, M.B., Cook, G.M., and Morales, S.E. (2016). Genomic and metagenomic surveys of hydrogenase distribution indicate H-2 is a widely utilised energy source for microbial growth and survival. *Isme Journal* 10**,** 761-777.

Happe, T., and Kaminski, A. (2002). Differential regulation of the Fe-hydrogenase during anaerobic adaptation in the green alga Chlamydomonas reinhardtii. *European Journal of Biochemistry* 269**,** 1022-1032.

Happe, T., and Naber, J.D. (1993). ISOLATION, CHARACTERIZATION AND N-TERMINAL AMINO-ACID-SEQUENCE OF HYDROGENASE FROM THE GREEN-ALGA CHLAMYDOMONAS-REINHARDTII. *European Journal of Biochemistry* 214**,** 475-481.

Kaji, M., Taniguchi, Y., Matsushita, O., Katayama, S., Miyata, S., Morita, S., and Okabe, A. (1999). The hydA gene encoding the H-2-evolving hydrogenase of Clostridium perfringens: molecular characterization and expression of the gene. *Fems Microbiology Letters* 181**,** 329-336.

Kamp, C., Silakov, A., Winkler, M., Reijerse, E.J., Lubitz, W., and Happe, T. (2008). Isolation and first EPR characterization of the FeFe -hydrogenases from green algae. *Biochimica Et Biophysica Acta-Bioenergetics* 1777**,** 410-416.

Kelly, C.L., Pinske, C., Murphy, B.J., Parkin, A., Armstrong, F., Palmer, T., and Sargent, F. (2015). Integration of an [FeFe]-hydrogenase into the anaerobic metabolism of Escherichia coli. *Biotechnology Reports* 8**,** 94-104.

Kertess, L., Adamska-Venkatesh, A., Rodriguez-Macia, P., Rudiger, O., Lubitz, W., and Happe, T. (2017). Influence of the 4Fe-4S cluster coordinating cysteines on active site maturation and catalytic properties of C. reinhardtii FeFe -hydrogenase. *Chemical Science* 8**,** 8127-8137.

King, P.W., Posewitz, M.C., Ghirardi, M.L., and Seibert, M. (2006). Functional studies of FeFe hydrogenase maturation in an Escherichia coli biosynthetic system. *Journal of Bacteriology* 188**,** 2163-2172.

Klein, M., Ansorge-Schumacher, M.B., Fritsch, M., and Hartmeier, W. (2010). Influence of hydrogenase overexpression on hydrogen production of Clostridium acetobutylicum DSM 792. *Enzyme and Microbial Technology* 46**,** 384-390.

Kpebe, A., Benvenuti, M., Guendon, C., Rebai, A., Fernandez, V., Le Laz, S., Etienne, E., Guigliarelli, B., Garcia-Molina, G., De Lacey, A.L., Baffert, C., and Brugna, M. (2018). A new mechanistic model for an O-2-protected electron-bifurcating hydrogenase, Hnd from Desulfovibrio fructosovorans. *Biochimica Et Biophysica Acta-Bioenergetics* 1859**,** 1302-1312.

Kuchenreuther, J.M., Grady-Smith, C.S., Bingham, A.S., George, S.J., Cramer, S.P., and Swartz, J.R. (2010). High-Yield Expression of Heterologous FeFe Hydrogenases in Escherichia coli. *Plos One* 5.

Lampret, O., Adamska-Venkatesh, A., Konegger, H., Wittkamp, F., Apfel, U.P., Reijerse, E.J., Lubitz, W., Rudiger, O., Happe, T., and Winkler, M. (2017). Interplay between CN- Ligands and the Secondary Coordination Sphere of the H-Cluster in FeFe -Hydrogenases. *Journal of the American Chemical Society* 139**,** 18222-18230.

Land, H., Ceccaldi, P., Meszaros, L.S., Lorenzi, M., Redman, H.J., Senger, M., Stripp, S.T., and Berggren, G. (2019). Discovery of novel FeFe -hydrogenases for biocatalytic H-2-production. *Chemical Science* 10**,** 9941-9948.

Land, H., Sekretareva, A., Huang, P., Redman, H.J., Nemeth, B., Polidori, N., Meszaros, L.S., Senger, M., Stripp, S.T., and Berggren, G. (2020). Characterization of a putative sensory FeFe -hydrogenase provides new insight into the role of the active site architecture. *Chemical Science* 11**,** 12789-12801.

Lautier, T., Ezanno, P., Baffert, C., Fourmond, V., Cournac, L., Fontecilla-Camps, J.C., Soucaille, P., Bertrand, P., Meynial-Salles, I., and Leger, C. (2011). The quest for a functional substrate access tunnel in FeFe hydrogenase. *Faraday Discussions* 148**,** 385-407.

Legall, J., Dervartanian, D.V., Spilker, E., Lee, J.P., and Peck, H.D. (1971). EVIDENCE FOR INVOLVEMENT OF NON-HEME IRON IN ACTIVE SITE OF HYDROGENASE FROM DESULFOVIBRIO-VULGARIS. *Biochimica Et Biophysica Acta* 234**,** 525-+.

Lemon, B.J., and Peters, J.W. (1999). Binding of exogenously added carbon monoxide at the active site of the iron-only hydrogenase (CpI) from Clostridium pasteurianum. *Biochemistry* 38**,** 12969-12973.

Leo, F., Schwarz, F.M., Schuchmann, K., and Muller, V. (2021). Capture of carbon dioxide and hydrogen by engineered Escherichia coli: hydrogen-dependent CO2 reduction to formate. *Applied Microbiology and Biotechnology* 105**,** 5861-5872.

Losey, N.A., Mus, F., Peters, J.W., Le, H.M., and Mcinerney, M.J. (2017). Syntrophomonas wolfei Uses an NADH-Dependent, Ferredoxin-Independent FeFe -Hydrogenase To Reoxidize NADH. *Applied and Environmental Microbiology* 83.

Losey, N.A., Poudel, S., Boyd, E.S., and Mcinerney, M.J. (2020). The Beta Subunit of Non-bifurcating NADH-Dependent FeFe -Hydrogenases Differs From Those of Multimeric Electron-Bifurcating FeFe -Hydrogenases. *Frontiers in Microbiology* 11**,** 14.

Malki, S., Saimmaime, I., Deluca, G., Rousset, M., Dermoun, Z., and Belaich, J.P. (1995). CHARACTERIZATION OF AN OPERON ENCODING AN NADP-REDUCING HYDROGENASE IN DESULFOVIBRIO FRUCTOSOVORANS. *Journal of Bacteriology* 177**,** 2628-2636.

Meszaros, L.S., Ceccaldi, P., Lorenzi, M., Redman, H.J., Pfitzner, E., Heberle, J., Senger, M., Stripp, S.T., and Berggren, G. (2020). Spectroscopic investigations under whole-cell conditions provide new insight into the metal hydride chemistry of FeFe -hydrogenase. *Chemical Science* 11**,** 4608-4617.

Meuser, J.E., Boyd, E.S., Ananyev, G., Karns, D., Radakovits, R., Murthy, U.M.N., Ghirardi, M.L., Dismukes, G.C., Peters, J.W., and Posewitz, M.C. (2011). Evolutionary significance of an algal gene encoding an FeFe -hydrogenase with F-domain homology and hydrogenase activity in Chlorella variabilis NC64A. *Planta* 234**,** 829-843.

Morra, S., Arizzi, M., Allegra, P., La Licata, B., Sagnelli, F., Zitella, P., Gilardi, G., and Valetti, F. (2014). Expression of different types of FeFe -hydrogenase genes in bacteria isolated from a population of a bio-hydrogen pilot-scale plant. *International Journal of Hydrogen Energy* 39**,** 9018-9027.

Morra, S., Arizzi, M., Valetti, F., and Gilardi, G. (2016a). Oxygen Stability in the New FeFe -Hydrogenase from Clostridium beijerinckii SM10 (CbA5H). *Biochemistry* 55**,** 5897-5900.

Morra, S., Cordara, A., Gilardi, G., and Valetti, F. (2015). Atypical effect of temperature tuning on the insertion of the catalytic iron-sulfur center in a recombinant FeFe -hydrogenase. *Protein Science* 24**,** 2090-2094.

Morra, S., Duan, J.F., Winkler, M., Ash, P.A., Happe, T., and Vincent, K.A. (2021). Electrochemical control of FeFe -hydrogenase single crystals reveals complex redox populations at the catalytic site. *Dalton Transactions* 50**,** 12655-12663.

Morra, S., Maurelli, S., Chiesa, M., Mulder, D.W., Ratzloff, M.W., Giamello, E., King, P.W., Gilardi, G., and Valetti, F. (2016b). The effect of a C298D mutation in CaHydA FeFe -hydrogenase: Insights into the protein-metal cluster interaction by EPR and FTIR spectroscopic investigation. *Biochimica Et Biophysica Acta-Bioenergetics* 1857**,** 98-106.

Morra, S., Mongili, B., Maurelli, S., Gilardi, G., and Valetti, F. (2016c). Isolation and characterization of a new FeFe -hydrogenase from Clostridium perfringens. *Biotechnology and Applied Biochemistry* 63**,** 305-311.

Mulder, D.W., Boyd, E.S., Sarma, R., Lange, R.K., Endrizzi, J.A., Broderick, J.B., and Peters, J.W. (2010). Stepwise FeFe -hydrogenase H-cluster assembly revealed in the structure of HydA(Delta EFG). *Nature* 465**,** 248-U143.

Mulder, D.W., Ratzloff, M.W., Bruschi, M., Greco, C., Koonce, E., Peters, J.W., and King, P.W. (2014). Investigations on the Role of Proton-Coupled Electron Transfer in Hydrogen Activation by FeFe -Hydrogenase. *Journal of the American Chemical Society* 136**,** 15394-15402.

Mulder, D.W., Ratzloff, M.W., Shepard, E.M., Byer, A.S., Noone, S.M., Peters, J.W., Broderick, J.B., and King, P.W. (2013). EPR and FTIR Analysis of the Mechanism of H-2 Activation by FeFe -Hydrogenase HydA1 from Chlamydomonas reinhardtii. *Journal of the American Chemical Society* 135**,** 6921-6929.

Nakos, G., and Mortenson, L. (1971). PURIFICATION AND PROPERTIES OF HYDROGENASE, AN IRON SULFUR PROTEIN, FROM CLOSTRIDIUM-PASTEURIANUM-W5. *Biochimica Et Biophysica Acta* 227**,** 576-+.

Nicolet, Y., De Lacey, A.L., Vernede, X., Fernandez, V.M., Hatchikian, E.C., and Fontecilla-Camps, J.C. (2001). Crystallographic and FTIR spectroscopic evidence of changes in Fe coordination upon reduction of the active site of the Fe-only hydrogenase from Desulfovibrio desulfuricans. *Journal of the American Chemical Society* 123**,** 1596-1601.

Nicolet, Y., Piras, C., Legrand, P., Hatchikian, C.E., and Fontecilla-Camps, J.C. (1999). Desulfovibrio desulfuricans iron hydrogenase: the structure shows unusual coordination to an active site Fe binuclear center. *Structure with Folding & Design* 7**,** 13-23.

Peters, J.W., Lanzilotta, W.N., Lemon, B.J., and Seefeldt, L.C. (1998). X-ray crystal structure of the Fe-only hydrogenase (Cpl) from Clostridium pasteurianum to 1.8 angstrom resolution. *Science* 282**,** 1853-1858.

Pohorelic, B.K.J., Voordouw, J.K., Lojou, E., Dolla, A., Harder, J., and Voordouw, G. (2002). Effects of deletion of genes encoding Fe-only hydrogenase of Desulfovibrio vulgaris Hildenborough on hydrogen and lactate metabolism. *Journal of Bacteriology* 184**,** 679-686.

Poudel, S., Tokmina-Lukaszewska, M., Colman, D.R., Refai, M., Schut, G.J., King, P.W., Maness, P.C., Adams, M.W.W., Peters, J.W., Bothner, B., and Boyd, E.S. (2016). Unification of FeFe -hydrogenases into three structural and functional groups. *Biochimica Et Biophysica Acta-General Subjects* 1860**,** 1910-1921.

Ratzloff, M.W., Artz, J.H., Mulder, D.W., Collins, R.T., Furtak, T.E., and King, P.W. (2018). CO-Bridged H-Cluster Intermediates in the Catalytic Mechanism of FeFe -Hydrogenase CaI. *Journal of the American Chemical Society* 140**,** 7623-7628.

Rodriguez-Macia, P., Galle, L.M., Bjornsson, R., Lorent, C., Zebger, I., Yoda, Y., Cramer, S.P., Debeer, S., Span, I., and Birrell, J.A. (2020). Caught in the H-inact: Crystal Structure and Spectroscopy Reveal a Sulfur Bound to the Active Site of an O-2-stable State of FeFe Hydrogenase. *Angewandte Chemie-International Edition* 59**,** 16786-16794.

Rodriguez-Macia, P., Pawlak, K., Rudiger, O., Reijerse, E.J., Lubitz, W., and Birrell, J.A. (2017). Intercluster Redox Coupling Influences Protonation at the H-cluster in FeFe Hydrogenases. *Journal of the American Chemical Society* 139**,** 15122-15134.

Rodriguez-Macia, P., Reijerse, E.J., Van Gastel, M., Debeer, S., Lubitz, W., Rudiger, O., and Birrell, J.A. (2018). Sulfide Protects FeFe Hydrogenases From O-2. *Journal of the American Chemical Society* 140**,** 9346-9350.

Roseboom, W., De Lacey, A.L., Fernandez, V.M., Hatchikian, E.C., and Albracht, S.P.J. (2006). The active site of the FeFe -hydrogenase from Desulfovibrio desulfuricans. II. Redox properties, light sensitivity and CO-ligand exchange as observed by infrared spectroscopy. *Journal of Biological Inorganic Chemistry* 11**,** 102-118.

Schuchmann, K., and Muller, V. (2012). A Bacterial Electron-bifurcating Hydrogenase. *Journal of Biological Chemistry* 287**,** 31165-31171.

Schuchmann, K., and Muller, V. (2013). Direct and Reversible Hydrogenation of CO2 to Formate by a Bacterial Carbon Dioxide Reductase. *Science* 342**,** 1382-1385.

Schut, G.J., and Adams, M.W.W. (2009). The Iron-Hydrogenase of Thermotoga maritima Utilizes Ferredoxin and NADH Synergistically: a New Perspective on Anaerobic Hydrogen Production. *Journal of Bacteriology* 191**,** 4451-4457.

Schwarz, F.M., Schuchmann, K., and Muller, V. (2018). Hydrogenation of CO2 at ambient pressure catalyzed by a highly active thermostable biocatalyst. *Biotechnology for Biofuels* 11**,** 11.

Senger, M., Mebs, S., Duan, J., Wittkamp, F., Apfel, U.P., Heberle, J., Haumann, M., and Stripp, S.T. (2016). Stepwise isotope editing of FeFe -hydrogenases exposes cofactor dynamics. *Proceedings of the National Academy of Sciences of the United States of America* 113**,** 8454-8459.

Shaw, A.J., Hogsett, D.A., and Lynd, L.R. (2009). Identification of the FeFe -Hydrogenase Responsible for Hydrogen Generation in Thermoanaerobacterium saccharolyticum and Demonstration of Increased Ethanol Yield via Hydrogenase Knockout. *Journal of Bacteriology* 191**,** 6457-6464.

Silakov, A., Kamp, C., Reijerse, E., Happe, T., and Lubitz, W. (2009). Spectroelectrochemical Characterization of the Active Site of the FeFe Hydrogenase HydA1 from Chlamydomonas reinhardtii. *Biochemistry* 48**,** 7780-7786.

Soboh, B., Linder, D., and Hedderich, R. (2004). A multisubunit membrane-bound NiFe hydrogenase and an NADH-dependent Fe-only hydrogenase in the fermenting bacterium Thermoanaerobacter tengcongensis. *Microbiology-Sgm* 150**,** 2451-2463.

Swanson, K.D., Ratzloff, M.W., Mulder, D.W., Artz, J.H., Ghose, S., Hoffman, A., White, S., Zadvornyy, O.A., Broderick, J.B., Bothner, B., King, P.W., and Peters, J.W. (2015). FeFe -Hydrogenase Oxygen Inactivation Is Initiated at the H Cluster 2Fe Subcluster. *Journal of the American Chemical Society* 137**,** 1809-1816.

Sybirna, K., Antoine, T., Lindberg, P., Fourmond, V., Rousset, M., Mejean, V., and Bottin, H. (2008). Shewanella oneidensis: a new and efficient system for expression and maturation of heterologous Fe-Fe hydrogenase from Chlamydomonas reinhardtii. *Bmc Biotechnology* 8.

Therien, J.B., Artz, J.H., Poudel, S., Hamilton, T.L., Liu, Z.F., Noone, S.M., Adams, M.W.W., King, P.W., Bryant, D.A., Boyd, E.S., and Peters, J.W. (2017). The Physiological Functions and Structural Determinants of Catalytic Bias in the FeFe -Hydrogenases CpI and CpII of Clostridium pasteurianum Strain W5. *Frontiers in Microbiology* 8**,** 11.

Vandijk, C., Mayhew, S.G., Grande, H.J., and Veeger, C. (1979). PURIFICATION AND PROPERTIES OF HYDROGENASE FROM MEGASPHAERA-ELSDENII. *European Journal of Biochemistry* 102**,** 317-330.

Verhagen, M., O'rourke, T., and Adams, M.W.W. (1999). The hyperthermophilic bacterium, Thermotoga maritima, contains an unusually complex iron-hydrogenase: amino acid sequence analyses versus biochemical characterization. *Biochimica Et Biophysica Acta-Bioenergetics* 1412**,** 212-229.

Vincent, K.A., Parkin, A., Lenz, O., Albracht, S.P.J., Fontecilla-Camps, J.C., Cammack, R., Friedrich, B., and Armstrong, F.A. (2005). Electrochemical definitions of O-2 sensitivity and oxidative inactivation in hydrogenases. *Journal of the American Chemical Society* 127**,** 18179-18189.

Von Abendroth, G., Stripp, S., Silakov, A., Croux, C., Soucaille, P., Girbal, L., and Happe, T. (2008). Optimized over-expression of FeFe hydrogenases with high specific activity in Clostridium acetobutylicum. *International Journal of Hydrogen Energy* 33**,** 6076-6081.

Voordouw, G., and Brenner, S. (1985). NUCLEOTIDE-SEQUENCE OF THE GENE ENCODING THE HYDROGENASE FROM DESULFOVIBRIO-VULGARIS (HILDENBOROUGH). *European Journal of Biochemistry* 148**,** 515-520.

Wang, S.N., Huang, H.Y., Kahnt, J., Mueller, A.P., Kopke, M., and Thauer, R.K. (2013a). NADP-Specific Electron-Bifurcating FeFe -Hydrogenase in a Functional Complex with Formate Dehydrogenase in Clostridium autoethanogenum Grown on CO. *Journal of Bacteriology* 195**,** 4373-4386.

Wang, S.N., Huang, H.Y., Kahnt, J., and Thauer, R.K. (2013b). A Reversible Electron-Bifurcating Ferredoxin- and NAD-Dependent FeFe -Hydrogenase (HydABC) in Moorella thermoacetica. *Journal of Bacteriology* 195**,** 1267-1275.

Winkler, M., Duan, J.F., Rutz, A., Felbek, C., Scholtysek, L., Lampret, O., Jaenecke, J., Apfel, U.P., Gilardi, G., Valetti, F., Fourmond, V., Hofmann, E., Leger, C., and Happe, T. (2021). A safety cap protects hydrogenase from oxygen attack. *Nature Communications* 12.

Winkler, M., Heil, B., and Happe, T. (2002). Isolation and molecular characterization of the Fe -hydrogenase from the unicellular green alga Chlorella fusca. *Biochimica Et Biophysica Acta-Gene Structure and Expression* 1576**,** 330-334.

Winkler, M., Senger, M., Duan, J.F., Esselborn, J., Wittkamp, F., Hofmann, E., Apfel, U.P., Stripp, S.T., and Happe, T. (2017). Accumulating the hydride state in the catalytic cycle of FeFe -hydrogenases. *Nature Communications* 8**,** 7.

Yang, S.H., Guarnieri, M.T., Smolinski, S., Ghirardi, M., and Pienkos, P.T. (2013). De novo transcriptomic analysis of hydrogen production in the green alga Chlamydomonas moewusii through RNA-Seq. *Biotechnology for Biofuels* 6**,** 17.

Zheng, Y.N., Kahnt, J., Kwon, I.H., Mackie, R.I., and Thauer, R.K. (2014). Hydrogen Formation and Its Regulation in Ruminococcus albus: Involvement of an Electron-Bifurcating FeFe -Hydrogenase, of a Non-Electron-Bifurcating FeFe -Hydrogenase, and of a Putative Hydrogen-Sensing FeFe -Hydrogenase. *Journal of Bacteriology* 196**,** 3840-3852.
